# Sirtuin 5 (SIRT5) Suppresses Tumor Growth by Regulating Mitochondrial Metabolism and Synaptic Remodeling in Gliomas

**DOI:** 10.3390/ijms25169125

**Published:** 2024-08-22

**Authors:** Wanjun Tang, Bo Chen, Gilberto Ka-Kit Leung, Karrie M. Kiang

**Affiliations:** 1 Department of Surgery, School of Clinical Medicine, LKS Faculty of Medicine, The University of Hong Kong, Hong Kong; 2 State Key Laboratory of Brain and Cognitive Sciences, The University of Hong Kong, Hong Kong

**Keywords:** glioma, sirtuin, SIRT5, glioblastoma, cancer metabolism, neuroplasticity, synapse modulation

## Abstract

Sirtuin 5 (SIRT5) is increasingly recognized as a key regulator of cellular metabolism, which is commonly dysregulated in cancer cells, resulting in enhanced proliferation and tumor progression. To investigate the clinicopathologic implications of SIRT5 dysregulation in glioblastoma, we performed comprehensive analyses of transcriptomic data and functional verifications using in vitro and in vivo glioblastoma models. We found that higher SIRT5 expression levels were associated with a favorable prognosis in glioma patients. Knockdown of SIRT5 significantly enhanced glioblastoma cell growth. Our data suggest its potential role in regulating mitochondrial metabolism in gliomas. Furthermore, SIRT5 is also significantly correlated with synaptic remodeling pathways. Our findings indicate a tumor-suppressive role for SIRT5 that extends beyond regulating cancer metabolism, by which it may function through modulating neuroplasticity. Understanding these cellular interactions provides nuanced insights into the multifaceted role of SIRT5 and the broader therapeutic implications of this for the development of novel treatment strategies.

## 1. Introduction

Gliomas, the most common primary brain tumors in adults, are classified by the World Health Organization (WHO) into Grades 1–4 lesions. Glioblastoma (GBM, Grade 4) has the worst prognosis, with a relative 5-year survival rate of approximately 6.9% [1]. Despite extensive studies, effective treatments for GBM remain elusive, underscoring the need for continued research. Recent advances in molecular profiling have enhanced our understanding of glioma biology and improved patient stratification based on biomarkers such as isocitrate dehydrogenase (IDH) mutation, 1p/19q codeletion, TERT promoter mutation, and O6-methylguanine-DNA methyltransferase (MGMT) promoter methylation [2]. IDH mutations and 1p/19q codeletion generally indicate better patient outcomes in oligodendrogliomas [3]. MGMT promoter methylation is predictive of the response to alkylating agents, particularly in GBM patients, and is associated with improved survival benefits [4]. These molecular markers also facilitate the identification of potential treatment targets in advancing translation research progress and personalized patient care strategies [5,6].

Sirtuins (SIRT1–7) are a family of NAD+-dependent deacetylases with diverse subcellular localizations and functions [7]. Recent studies have implicated sirtuins in various physiological and pathological processes, including aging [8] and neurodegenerative disorders [9]. However, their role in cancer remains unclear [10], with SIRT5 probably being the least understood in the context of tumor biology. SIRT5 is primarily localized in the mitochondria and has been shown to orchestrate diverse metabolic processes through its multifaceted enzymatic activities, including the deacetylation [11], decarbonylation, desuccinylation [12], and deglutarylation [13] of key metabolic proteins. In pancreatic ductal adenocarcinoma (PDAC) cells, for example, SIRT5 catalyzes the deacetylation of aspartate transaminase 1 (GOT1) at specific lysine residues, thereby inhibiting its enzymatic activity and suppressing the glutamine–glutathione metabolic pathway. Consequently, the suppression of SIRT5 could enhance glutamine metabolism and the generation of glutathione and NADPH to maintain redox balance and promote cell proliferation in PDAC cells [14]. A few studies have also suggested that SIRT5 expression may be reduced in glioma samples [15], with higher expression levels associated with improved patient survival [16].

The present study aimed to investigate the functional role of SIRT5 in glioma pathogenesis. We found that downregulated SIRT5 expression was strongly correlated with a poorer patient prognosis, while a loss of SIRT5 function significantly enhanced GBM cell proliferation both in vitro and in vivo, possibly by disrupting mitochondrial signaling pathways. Notably, we uncovered a profound association between SIRT5 expression and the molecular pathways governing neuroplasticity, suggesting a novel avenue for future research into the interplay between SIRT5 and the tumor microenvironment.

## 2. Results

### 2.1. SIRT5 Expression Correlates with Better Survival in Glioma Patients

**Figure 1:**
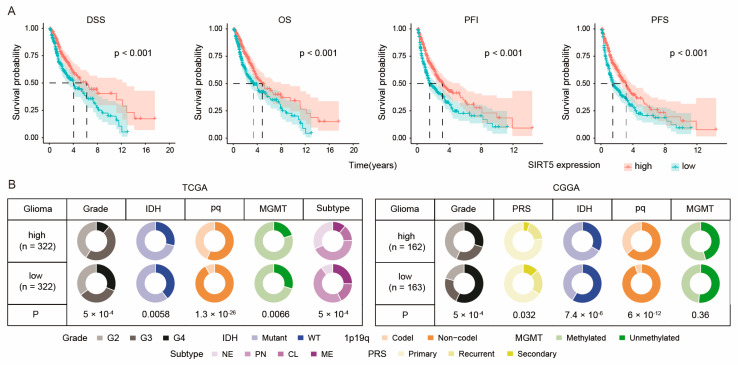
SIRT5 expression is related to better survival among glioma patients as well as to tumor grading. (**A**) Kaplan–Meier survival curves comparing DSS, OS, PFI, and PFS between patients with high- and low-SIRT5-expression gliomas from the TCGA dataset (number of high-SIRT5 patients = 322; number of low-SIRT5 patients = 322). DSS, disease-specific survival; OS, overall survival; PFI, progression-free interval; PFS, progression-free survival. (**B**) Clinicopathologic features between patients from the high- and low-SIRT5 groups from the TCGA and CGGA datasets. Tumor grade (G2, Grade 2; G3, Grade 3; G4, Grade 4), IDH mutation status (WT, wildtype), 1p19q (codel, codeletion; non-codel, non-codeletion), molecular subtypes (NE, neural; PN, proneural; CL, classic; ME, mesenchymal), PRS (primary, recurrent, secondary).

We first investigated the clinical significance of SIRT5 in glioma patients. Our analysis revealed that high SIRT5 expression was associated with improved patient outcomes, including a longer duration of disease-specific survival (DSS), overall survival (OS), progression-free interval (PFI), and progression-free survival (PFS) in Grade 2–4 glioma patients (Figure 1A). Furthermore, SIRT5 expression levels were inversely correlated with the degree of tumor malignancy (Figure 1B). Notably, SIRT5 was downregulated in recurrent and secondary gliomas, as well as gliomas with specific genetic characteristics, such as the IDH wildtype, unmethylated MGMT, and 1p19q non-codeletion (Figure 1B). Additionally, we found that SIRT5 expression was significantly downregulated in the more aggressive classic (CL) and mesenchymal (ME) molecular subtypes of glioma compared to the proneural (PN) and neural (NE) subtypes [17] (Figure 1B), suggesting a correlation between low SIRT5 expression levels and aggressive tumor characteristics. These findings suggest that SIRT5 expression is clinically significant in gliomas, correlating with both patient prognosis and tumor grading.

### 2.2. SIRT5 Knockdown Promotes GBM Cell Growth In Vitro and In Vivo

**Figure 2:**
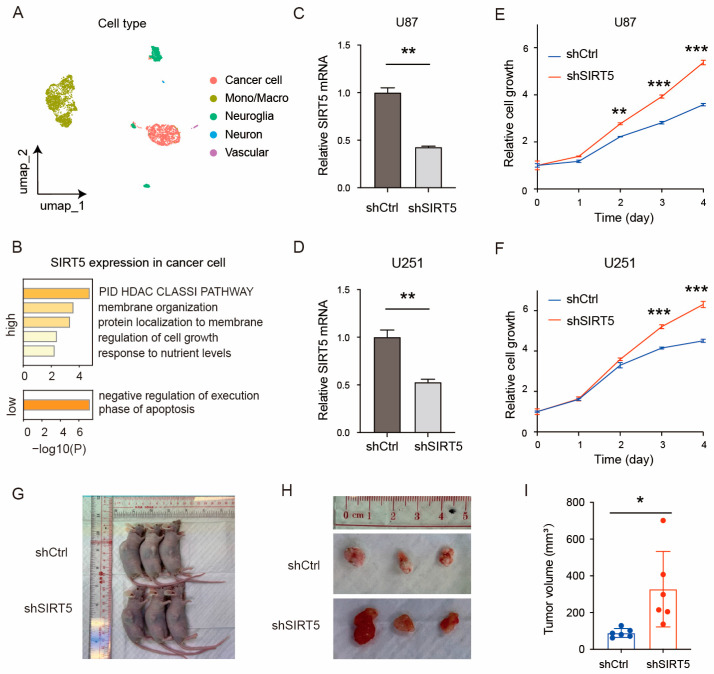
SIRT5 knockdown promotes GBM cell growth in vitro and in vivo. (**A**) Annotation of cell types in the single-cell dataset. (**B**) GO functional enrichment analysis of differentially expressed genes between high- and low-SIRT5-expression cancer cells. (**C**,**D**) Quantification of SIRT5 mRNA expression in U87 (**C**) and U251 (**D**) GBM cells expressing shRNA targeting SIRT5 (shSIRT5) or a control shRNA (shCtrl). Data are presented as the mean ± SD (*n* = 3 per group). (**E**,**F**) Cell proliferation assay showing the proliferation of U87 (**E**) and U251 (**F**) GBM cells expressing shSIRT5 or shCtrl. Data are presented as the mean ± SD (*n* = 5 per condition). (**G**) Representative image of subcutaneous xenograft tumors formed by U87 cells expressing shSIRT5 or shCtrl 21 days post injection in nude mice. (**H**,**I**) Quantification of tumor volume in subcutaneous xenograft experiments using U87 cells expressing shSIRT5 and shCtrl 21 days post injection. Data are presented as the mean ± SD (*n* = 6 per group). Statistical significance was determined using Student’s *t*-test: * *p* < 0.05; ** *p* < 0.01; *** *p* < 0.001.

To investigate the biological function of SIRT5 in glioma, we performed a series of experiments using single-cell analysis, in vitro assays, and in vivo xenograft studies. Single-cell RNA sequencing analysis of glioma samples identified five major cell types: cancer cells, monocytes/macrophages, neuroglia cells, neurons, and vascular cells (Figure 2A). GO enrichment analysis revealed that genes enriched in high-SIRT5 cancer cells were involved in the regulation of cell growth and membrane organization (Figure 2B). To determine the role of SIRT5 in GBM cell growth, we knocked down SIRT5 expression using shRNA in the U87 (Figure 2C) and U251 (Figure 2D) GBM cell lines. Cell proliferation assays showed that this SIRT5 knockdown (shSIRT5) significantly enhanced the growth of both U87 (Figure 2E) and U251 (Figure 2F) cells compared to the control group (shCtrl). In vivo xenograft experiments further demonstrated that tumors derived from SIRT5-knockdown U87 cells grew to significantly larger volumes than controls at 3 weeks post implantation in mice (Figure 2G–I). Collectively, these results suggest that SIRT5 functions as a tumor suppressor in GBM, and its loss of function promotes GBM cell proliferation in vitro and tumor growth in vivo.

### 2.3. SIRT5 Potentially Regulates GBM Cell Growth through Modulation of Mitochondrial Pathways

**Figure 3:**
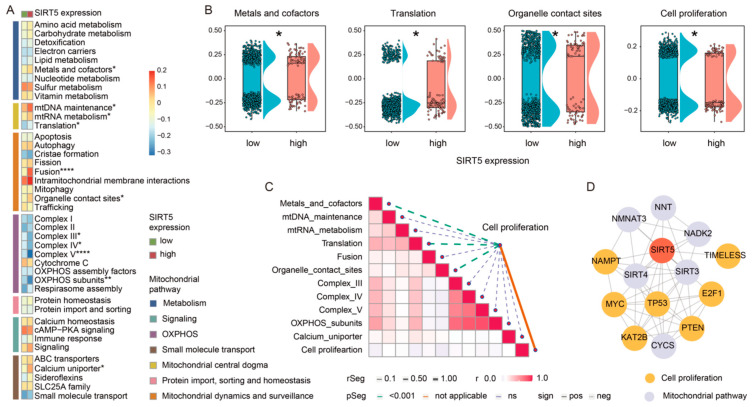
SIRT5 potentially regulates GBM cell growth through the modulation of mitochondrial pathways. (**A**,**B**) Comparison of mitochondrial pathway gene expression between high- and low-SIRT5-expression cancer cells in the TCGA dataset. (**C**) Correlation analysis between mitochondrial pathway activity scores and cell proliferation levels in the TCGA dataset. (**D**) Protein–protein interaction network depicting the connections between SIRT5, key mitochondrial pathway proteins (e.g., NNT and SIRT3), and cell proliferation regulators (e.g., E2F1 and TIMELESS). * *p* < 0.05; ** *p* < 0.01; **** *p* < 0.0001.

SIRT5 is primarily localized within mitochondria [18]. To investigate how SIRT5 inhibition promotes GBM cell growth, we performed a bioinformatics analysis of mitochondrial pathway activity. Comparative analysis of the mitochondrial pathway gene expression revealed 11 pathways that were significantly different between high- and low-SIRT5-expression cancer cells (Figure 3A,B). Notably, three mitochondrial pathways—metals and cofactors (mitochondrial metabolism), translation (mitochondrial central dogma), and organelle contact sites (mitochondrial dynamics and surveillance)—showed a strong negative correlation with cell proliferation levels (Figure 3C). Further regulatory network analysis suggested close protein interactions among these mitochondrial pathways, SIRT5, and key cell proliferation regulators (Figure 3D). Based on these findings, we speculate that SIRT5 may regulate GBM cell proliferation, at least in part, through its modulation of specific mitochondrial pathways involved in metabolism, translation, and mitochondrial dynamics.

### 2.4. SIRT5 May Regulate Synapse Function and Immune Response in Gliomas

**Figure 4:**
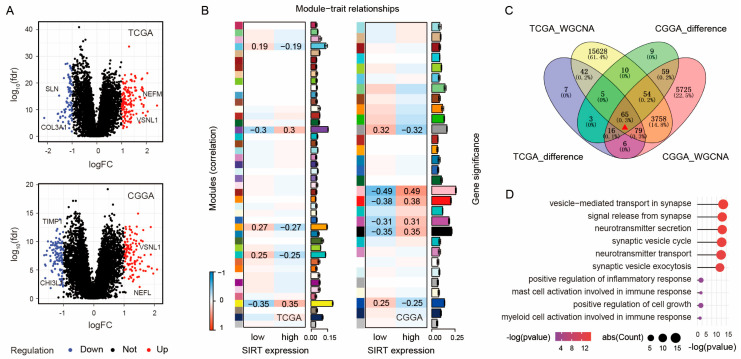
SIRT5 may regulate synapse function and immune response in gliomas. (**A**) Volcano plots displaying differentially expressed genes between high- and low-SIRT5-expression gliomas in the TCGA and CGGA datasets. (**B**) WGCNA plots showing the modules of genes highly correlated with SIRT5 expression levels in the TCGA and CGGA glioma samples. (**C**) Venn diagram depicting the overlap (indicated by red triangle) between differentially expressed genes and genes highly correlated with SIRT5 expression based on the WGCNA results. (**D**) GO functional enrichment analysis of the 65 genes that were both differentially expressed and highly correlated with SIRT5 levels. Only the top ten most enriched biological processes are shown.

To further elucidate the biological functions of SIRT5 in gliomas, we conducted a comprehensive analysis of gene expression data from bulk tumor samples. We separately identified differentially expressed genes (DEGs) between high- and low-SIRT5 gliomas in the TCGA and CGGA datasets. In TCGA, we found 223 upregulated genes and 221 downregulated genes (log2FC > 1.0 or log2FC < −1.0, and FDR < 0.05), including highly upregulated genes like NEFM and VSNL1 and highly downregulated genes such as SLN and COL3A1 (Figure 4A). Similarly, the CGGA dataset revealed 113 upregulated genes and 108 downregulated genes, including highly upregulated genes such as VSNL1 and NEFL and highly downregulated genes such as TIMP1 and CHI3L (Figure 4A). Next, WGCNA was used to identify gene modules highly correlated with SIRT5 expression in the TCGA and CGGA glioma samples. In TCGA, we identified 19,851 module genes that were highly related to SIRT5 expression, while in CGGA, we found 8442 such genes (Figure 4B). By intersecting the DEGs and WGCNA-identified genes, we obtained 65 genes that were both differentially expressed and highly correlated with SIRT5 levels (Figure 4C). GO enrichment analysis of these genes revealed that they were involved in synapse organization, immune response, and the regulation of cell growth (Figure 4D). Finally, after intersection analysis, we obtained 65 genes that were both differentially expressed and highly correlated with the SIRT5 expression level (Figure 4C). Subsequent GO enrichment indicated that the biological function of these genes focused on synapse transport, immune response, and cell growth regulation (Figure 4D). Collectively, these results suggest that SIRT5 may regulate not only glioma cell growth but also the activities of synapses and immune responses in gliomas. These findings underscore the need for further investigation to validate these observations and explore the potential applications for advancing our understanding of glioma biology.

## 3. Discussion

SIRT5 exhibits context-dependent dual effects in cancers by acting as either a tumor suppressor or an oncogenic factor [19]. Previous studies have demonstrated SIRT5’s tumor-suppressive roles in PDAC [14] and renal cell carcinoma [20], while others have reported an oncogenic function in breast cancers [21,22]. Given these findings, we sought to elucidate the clinical significance and functional role of SIRT5 in gliomas, with an emphasis on its effect on biological pathways. Our investigations revealed that decreased SIRT5 expression correlates with poorer glioma patient survival. Single-cell analysis indicated a correlation between SIRT5 expression and cell growth regulation. Experimental validation demonstrated SIRT5’s capacity to suppress GBM cell growth. Collectively, our findings strongly suggest a tumor-suppressive function of SIRT5 in gliomas.

The strong association between low SIRT5 expression and aggressive glioma characteristics suggests that SIRT5 could serve as a valuable biomarker for assessing glioma grades. Recent technological advancements have enabled the detection of biomarker levels [23] in small populations of cells from glioma specimens [24] or patient blood samples [25]. These innovations could lead to more precise and convenient diagnostic methods, enhancing the utility of SIRT5 expression in the diagnosis and prognostic prediction of gliomas. Mechanistically, we hypothesized that SIRT5 exerts tumor-suppressive effects primarily through the regulation of mitochondrial metabolism. As one of the mitochondrial sirtuins, along with SIRT3 and SIRT4, SIRT5 modulates energy metabolism through the post-translational modifications of target proteins [26]. Notably, SIRT5 is currently being recognized as the primary enzyme catalyzing lysine desuccinylation and demalonylation, with succinylation being a widespread post-translational modification among mitochondrial metabolic enzymes [27]. Previous studies have demonstrated that SIRT5 modulates mitochondrial respiration in mouse embryonic fibroblasts by regulating the activity of two crucial enzyme complexes: the pyruvate dehydrogenase complex (PDC) and succinate dehydrogenase (SDH). This regulation occurs primarily through SIRT5’s ability to remove succinyl groups from specific lysine residues on these target proteins [28]. These enzyme complexes play pivotal roles in mitochondrial metabolism, with PDC linking glycolysis to the tricarboxylic acid (TCA) cycle and SDH functioning in both the TCA cycle and the electron transport chain. Additionally, SIRT5 helps maintain mitochondrial integrity and function by modulating mitochondrial morphology and dynamics [29]. Our bioinformatics analysis further supports and extends these findings regarding the regulatory role of SIRT5 in mitochondrial metabolism. We observed that SIRT5 expression is strongly correlated with mitochondrial metabolism, mitochondrial dynamics and surveillance, and oxidative phosphorylation-related pathways, which is consistent with previous reports showing its aberrant regulation of lysine succinylation in various cancers [30]. Future studies are needed to identify SIRT5 target proteins in gliomas and how these mitochondrial modifications may impact metabolism and energy homeostasis. Emerging evidence also demonstrates the crucial role of SIRT5-mediated lysine desuccinylation in modulating cancer immunity [31]. Specifically, SIRT5 may prevent tumor immune evasion and suppress hepatocellular carcinoma development [32]. Our findings suggest a possible link between SIRT5 expression and immune response regulation in gliomas. This association may be mediated through the modulation of mast cell and myeloid cell activation, both of which play critical roles in the immune response. To fully comprehend the role of SIRT5 and its implications for research and therapeutics, future mechanistic studies are crucial to define how SIRT5 regulates these specific processes.

Intriguingly, our bioinformatics analysis of public datasets revealed a potential role for SIRT5 in regulating synaptic functions. We observed a significant correlation between SIRT5 expression and various pathways related to synapse formation and functioning. While direct evidence linking SIRT5 to synaptic plasticity is limited, its regulatory role in mitochondrial function suggests that it may influence synaptic integrity and plasticity indirectly. By modulating mitochondrial dynamics, SIRT5 may support the maintenance of synaptic function and connections, thereby playing a role in synaptic dynamics [33]. These findings are noteworthy given the emerging importance of synaptic interactions in glioma pathogenesis [34]. Glioma cells engage in complex interactions with surrounding cells [35], such as neurons [36], via synaptic connections. While the regulatory effects of other sirtuins, such as SIRT1 [37] and SIRT3 [38], on synaptic plasticity have been identified, the role of SIRT5 in this process remains largely unexplored. VSNL1, the gene highly upregulated upon high SIRT5 expression in gliomas (Figure 4A), encodes visinin-like protein 1 (VILIP-1). This protein is involved in several pathways related to synaptic function and plasticity [39] and its presence has been found to be decreased in glioma samples [40]. The underlying mechanism behind this positive correlation between SIRT5 and VSNL1 expression in glioma samples deserves further investigation by utilizing, for example, transgenic SIRT5-knockout mouse models to examine the interactions between SIRT5 and synapse-related pathways and its impacts on glioma development and invasion [41].

In conclusion, this study advances our understanding of SIRT5’s role in glioma biology and its potential as a prognostic biomarker. Our findings underscore the necessity for further in-depth investigation into the diverse functions of SIRT5 in glioma progression, particularly its impacts on mitochondrial metabolism, immune modulation, and synaptic remodeling. Such investigations could provide invaluable insights into the mechanisms underlying glioma pathogenesis and inform the development of effective therapeutic strategies.

## 4. Materials and Methods

### 4.1. Sample and Data Collection

We collected the transcriptome data for gliomas from the Cancer Genome Atlas (TCGA, https://portal.gdc.cancer.gov/, accessed on 11 August 2022), China Glioma Genome Atlas (CGGA, http://www.cgga.org.cn/ accessed on 11 August 2022), and Gene Expression Omnibus (GEO, GSE84465, https://www.ncbi.nlm.nih.gov/geo/ accessed on 11 August 2022) databases. A total of 644 bulk glioma transcriptome samples were included in the TCGA cohort, with 325 bulk glioma transcriptome samples in the CGGA cohort and 3533 GBM single-cell transcriptome cells in the GEO cohort.

### 4.2. Bulk Transcriptome Data Analysis

The 50% median value of SIRT5 mRNA expression was set as the cutoff to divide bulk samples into low- and high-SIRT5 groups. The Wilcoxon rank sum test was applied to compute the differentially expressed genes, and weighted gene co-expression network analysis (WGCNA) was used to screen high-correlation genes. Next, the differentially expressed genes and high-correlation genes were intersected. Potential biological functions were annotated using gene ontology (GO) terms.

### 4.3. Single-Cell Transcriptome Data Analysis

The single-cell data were processed using the Seurat R package (version 5.0.1). Cell types were annotated according to a previous study [42]. The cancer cells were divided into low- and high-SIRT5 groups according to the 90% quantile value of SIRT5 expression, which was determined based on the proportion of cancer cells without SIRT5 expression. Next, the function “FindMarkers” was used to assess the differentially expressed genes. GO terms and mitochondrial pathways [43,44] were applied to annotate biological functions. The protein–protein interaction network was generated using the “String” tool [45].

### 4.4. Cell Culture

Human glioma cell lines U87MG (U87) and U251MG (U251) were purchased from the American Type Culture Collection (ATCC, Manassas, VA, USA). Both cell lines were cultured in Minimum Essential Medium Alpha (MEM-α) (Gibco, Waltham, MA, USA), supplemented with 10% fetal bovine serum (Gibco) and 1% penicillin streptomycin (Gibco). The cells were maintained at 37 °C in a 5% CO_2_ and 95% air incubator.

### 4.5. Transfection of Short Hairpin RNA (shRNA)

shRNA was designed and assessed according to the method proposed by Moore et al. [46]. Lentiviral vectors (pGLVU6/Firefly/Puro) with shRNA targeting human SIRT5 (shSIRT5) and scramble control (shCtrl) were purchased from Genepharma (Suzhou, China). Lentivirus packaging in HEK293T cells was conducted using Lenti-X Packaging Single Shots (VSV-G) (Takara, Bio, Japan). Lentiviral supernatants were collected at 48 h and 72 h post transfection and filtered according to the manufacturer’s protocol. U87 and U251 cells were infected with lentivirus-containing media with polybrene (Sigma-Aldrich, St. Louis, MO, USA) supplemented at 5 μg/mL for 48 h. Cell clones were selected using 2 μg/mL puromycin.

### 4.6. Real-Time Quantitative PCR (RT-qPCR)

Total RNA was extracted using TRIzol reagent (Invitrogen, Carlsbad, CA, USA). The synthesis of cDNA from RNA was performed using a PrimeScript RT Reagent Kit (Takara), according to the manufacturer’s protocol. Then, qPCR was performed to quantify the mRNA expression of SIRT5 and GAPDH using the SYBR Green-based PCR kit (Takara). The following primers were used for the detection of SIRT5: forward: 5′- AGGAAAAGGGTGTGAAGAGGC -3′; reverse: 5′- GGAAGTGCCCACCACTAGAC -3′. For the detection of GAPDH, the primers were as follows: forward: 5′- GCTCTCTGCTCCTCCTGTTC -3′; reverse: 5′- ACGACCAAATCCGTTGACTC -3′. The ViiA-7 Real-Time PCR system (Applied Biosystems, Foster City, CA, USA) was utilized to perform the real-time PCR reaction, and the data were analyzed using the 2^−ΔΔCT^ method after normalization with GAPDH gene expressions [47].

### 4.7. Cell Proliferation Assay

Cell proliferation was measured with the sulforhodamine B (SRB) assay according to a previously published protocol [48]. Cells were seeded onto 96-well plates at a density of 2000 cells/well, with five replicates per condition. The total amount of protein was determined at days 0, 1, 2, 3, and 4 after cell adhesion.

### 4.8. Subcutaneous Xenograft Model

Male BALB/c-nu/nu athymic nude mice (6–8 weeks old) were obtained from the Laboratory Animal Unit of the University of Hong Kong. The mice were randomized to inoculate 1 × 10^6^ U87 cells expressing either shCtrl or shSIRT5. The cells were injected subcutaneously into the right flank of each mouse. Tumor volume was recorded twice a week, and the tumor xenografts were collected 3 weeks post cell injection. All animal experiments were conducted in compliance with the Animals (Control of Experiments) Ordinance of Hong Kong. The experimental procedures were approved and conducted under the Committee on the Use of Live Animals for Teaching and Research (CULATR) guidelines. All mice were housed in a temperature-controlled environment with a 12 h light/dark cycle, with unrestricted access to food and water. Mice were monitored daily for their health and welfare.

### 4.9. Statistical Analyses

Statistical analyses were conducted using the software R (version 4.3.1) and GraphPad Prism (version 8). The two-tailed Wilcoxon test and Fisher’s exact test were used to compare continuous variables and categorical variables, respectively. Spearman correlation analysis and Kaplan–Meier survival analysis were also applied. Data for in vitro and in vivo experiments were plotted as the mean ± standard deviation (SD). Student’s *t*-test was used to compare the two groups. *p*-values less than 0.05 were considered statistically significant.

## Data Availability

The original contributions presented in the study are included in the article; any further inquiries can be directed to the corresponding authors.
